# Gas6/MerTK signaling is negatively regulated by NF-κB and supports lung carcinogenesis

**DOI:** 10.18632/oncotarget.27345

**Published:** 2019-12-17

**Authors:** Sergey V. Novitskiy, Rinat Zaynagetdinov, Georgii Vasiukov, Sergey Gutor, Wei Han, Ana Serezani, Anton Matafonov, Linda A. Gleaves, Taylor P. Sherrill, Vasiliy V. Polosukhin, Timothy S. Blackwell

**Affiliations:** ^1^ Division of Allergy, Pulmonary and Critical Care Medicine, Department of Medicine, Vanderbilt University, Nashville, TN 37212, USA; ^2^ Department of Pathology, Microbiology, and Immunology, Vanderbilt University, Nashville, TN 37212, USA; ^3^ Department of Cell and Developmental Biology, Vanderbilt University Medical Center, Nashville, TN 37212, USA; ^4^ Department of Veterans Affairs Medical Center, Nashville, TN 37212, USA

**Keywords:** lung cancer, NF-κB, Mer-TK, macrophage, Kras

## Abstract

Growth arrest-specific 6 (Gas6) has been implicated in carcinogenesis through activation of its receptors, particularly MerTK. To investigate whether Gas6 plays a role in resistance to NF-κB inhibitors, which have not proven to be effective agents for lung cancer therapy, we studied lung cancer models induced by urethane injection or expression of mutant Kras (Kras^G12D^). We found that Gas6 is primarily produced by macrophages during tumorigenesis and that Gas6 is negatively regulated by NF-κB. Since Gas6 is a vitamin K dependent protein, we used low-dose warfarin to block Gas6 production and showed that this treatment inhibited tumorigenesis in both the urethane and Kras^G12D^ models, most prominently in mice with targeted deletion of IKKβ in myeloid cells (IKKβ^ΔMye^ mice). In addition, MerTK deficient mice had reduced urethane-induced tumorigenesis. Inhibition of the Gas6-MerTK pathway in all these models reduced macrophages and neutrophils in the lungs of tumor-bearing mice. Analysis of mouse lung tumors revealed MerTK staining on tumor cells and *in vitro* studies showed that Gas6 increased proliferation of human lung cancer cell lines. To assess the therapeutic potential for combination treatment targeting NF-κB and Gas6-MerTK, we injected Lewis Lung Carcinoma cells subcutaneously and treated mice with Bay 11-70852 (NF-κB inhibitor) and/or Foretinib (MerTK inhibitor). While individual treatments were ineffective, combination therapy markedly reduced tumor growth, blocked tumor cell proliferation, reduced tumor-associated macrophages, and increased CD4+ T cells. Together, our studies unmask a role for Gas6-MerTK signaling in lung carcinogenesis and indicate that up-regulation of Gas6 production in macrophages could be a major mechanism of resistance to NF-κB inhibitors.

## INTRODUCTION

Lung cancer is the leading cause of cancer death in the United States [1]. Among the inflammatory cell types infiltrating the lungs during carcinogenesis, macrophages are the most heterogeneous, displaying phenotypic plasticity depending on the microenvironment. We and others previously demonstrated that macrophages support lung tumor promotion and growth in animal models [2–6]. In addition, there is a strong correlation between macrophage density in tumors, microvessel counts, and relapse-free survival in humans with lung cancer [7].

NF-κB has been identified as a central pathway that controls innate immunity and inflammatory responses through production of pro-inflammatory cytokines and chemokines, enzymes, and regulators of cell survival/apoptosis. Accumulating evidence demonstrates that activation of NF-κB is associated with carcinogenesis and plays an important role in malignant cell survival and proliferation [8]. Inhibition of NF-κB in airway epithelial cells significantly reduces lung tumorigenesis in mice [9–12]. Consistent with a critical role for epithelial NF-κB in lung tumorigenesis, we showed that transgenic mice with inducible expression of a constitutively active form of IKKβ develop 3-4-fold more lung tumors after carcinogen treatment [13]. These data point to NF-κB inhibition as a potentially effective strategy for prevention or treatment of lung cancer. However, using a genetic model of lung carcinogenesis (Kras^G12D^), Xue et al. demonstrated that systemic treatment with NF-κB inhibitors was ineffective at long-term reduction of tumor volume [14]. While treatment with NF-κB inhibitors initially reduced tumor size and tumor cell proliferation, this effect was short-lived. Consistent with this report, we showed that prolonged NF-κB inhibitor treatment in mice resulted in increased lung tumor formation after treatment with urethane [15].

Tyro3-Axl-MerTK (TAM) receptors (TAMR) represent a family of receptor tyrosine kinases that support cell survival, proliferation, migration, and differentiation [16]. Increased expression of TAMR members has been detected in tumors, including in the lung, and correlates with poor prognosis [16–18]. Inhibition of MerTK in non-small cell lung cancer (NSCLC) has been demonstrated to decrease colony formation *in vitro* and reduce growth of subcutaneous xenografts in nude mice [16]. Two ligands for TAMRs are known: Gas6 and Protein S. These proteins share 42% amino acid homology and consist of a vitamin K-dependent N-terminal gamma-carboxylated glutamic acid domain followed by 4 EGF-like domains and 2 C-terminal globular laminin G-like domains [19]. While Protein S is a constitutively produced plasma protien, Gas6 is typically present in subnanomolar amounts [20], but its production is substantially increased in a variety of human tumors [21]. Higher levels of Gas6 correlate with increased mortality of cancer patients [22, 23]. Pro-oncogenic effects of Gas6, including increased cell survival and proliferation, are transduced through interactions with TAM receptors, particularly MerTK [15, 17]. In human lung tumor cell lines, activation of MerTK by Gas6 has been shown to induce phosphorylation of Erk1/2 and PI3K/Akt [16, 17, 24]. In models of colorectal and breast cancers, Loges et al. demonstrated that macrophages represent the main source of Gas6 in the tumor microenvironment and genetic deletion of Gas6 attenuates tumor growth [25]. Gas6 expression has been reported to inversely correlate with NF-κB activity in peritoneal macrophages [26]; therefore, we postulated that global inhibition of NF-κB signaling could result in increased Gas6 expression by macrophages, thereby mitigating the beneficial effects of NF-κB inhibition in tumor cells.

In this study, we investigated an association between the TAMR pathway and NF-κB signaling during lung carcinogenesis in Kras^G12D^ and urethane models. Inhibition of Gas6 or deletion of MerTK blocked lung tumor formation, particularly in the setting of NF-κB inhibition. Further studies suggested that systemic treatment using a combination of MerTK and NF-κB inhibition could be effective for decreasing tumor growth.

## RESULTS

### NF-κB down-regulates Gas6 in myeloid cells

**Figure 1:**
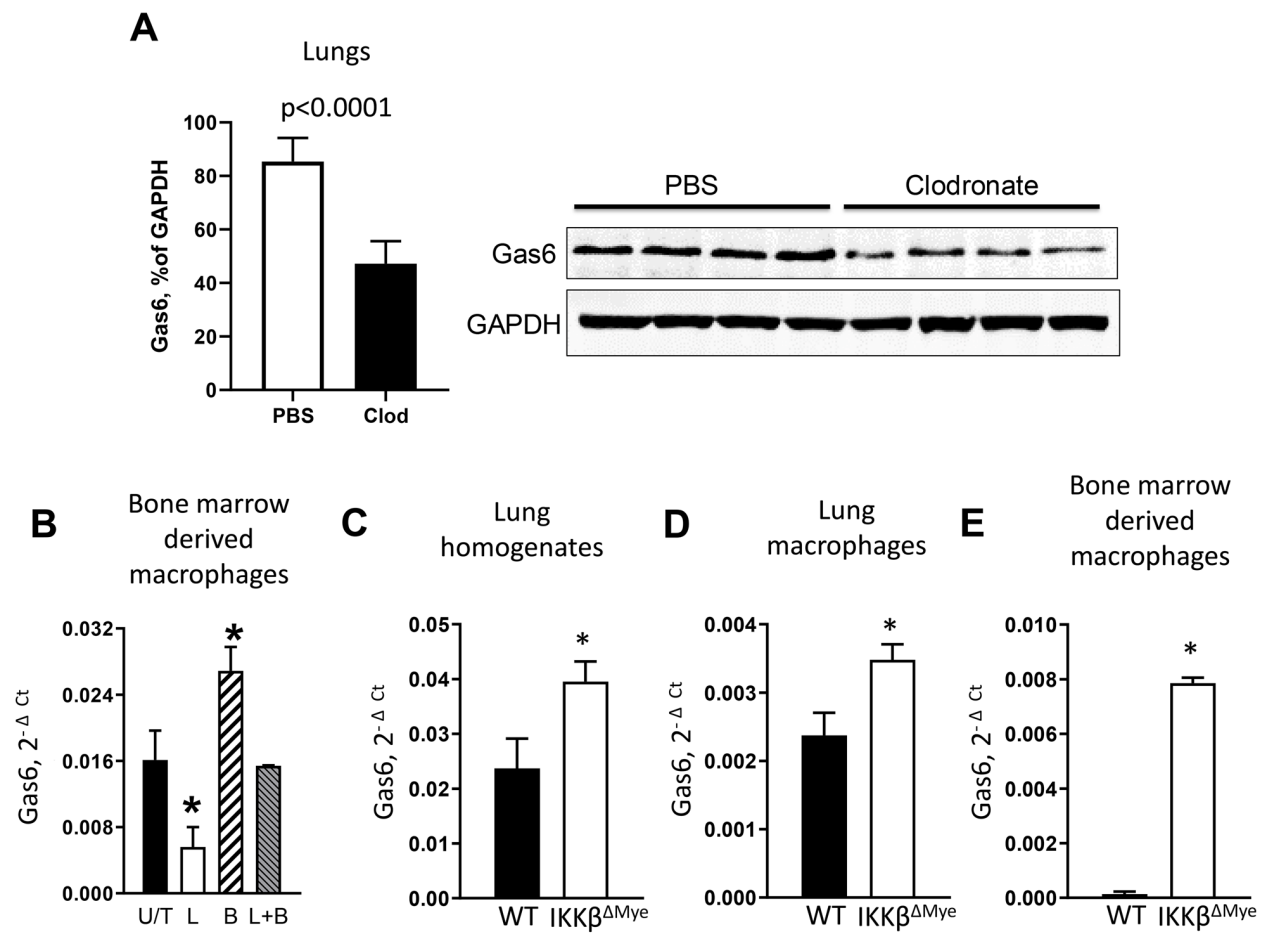
NF-κB down-regulates Gas6 expression in myeloid cells **(A)** Expression of Gas6 by western blot (normalized to GAPDH) in lungs from WT mice treated with intratracheal clodronate (Clod) or empty (PBS) liposomes on day 0 and day 7 after urethane injection. Lungs were harvested on day 14. **(B)** mRNA expression of Gas6 in bone marrow-derived macrophages from WT mice that were untreated (U/T) or treated with LPS (L, 100 ng/ml) or/and Bay-117082 (B, 10 uM) for 4 hours. ^*^p<0.05 compared to U/T cells. **(C)** mRNA expression of Gas6 in lung homogenates and **(D)** lung macrophages from WT or IKKβ^ΔMye^ mice at Day 7 after single injection of urethane (n=3 per group). **(E)** Gas6 mRNA expression in bone marrow-derived macrophages from WT and IKKβ^ΔMye^ mice after 48-hour incubation in DMEM media (+ 10% FBS) supplemented with 30% conditioned medium from Lewis Lung Carcinoma cells, ^*^ p<0.05 compared to WT. Data correspond to the mean ± SEM, n=5 mice/group.

In initial studies, we investigated whether lung macrophages express Gas6 during lung tumorigenesis. We injected wild type (WT) mice with urethane (1 g/kg) by intraperitoneal injection, followed by intratracheal (IT) treatment with liposomal clodronate to deplete macrophages on day 0 and day 7 post-urethane. At day 14 after urethane, macrophage depleted mice showed a marked reduction in Gas6 expression in the lungs (Figure 1A). Next, we investigated whether activation of NF-κB could reduce expression of Gas6 in macrophages. Bone marrow-derived macrophages from WT mice were treated with *E. coli* lipopolysaccharide (LPS) to activate NF-κB and 4 hours later cells were harvested for analysis of Gas6 mRNA expression. As shown in Figure 1B, activation of NF-κB in macrophages significantly reduced Gas6 mRNA, which was restored after co-incubation in the presence of a NF-κB inhibitor, Bay-117082.

To investigate the connection between Gas6 and NF-κB activation *in vivo*, we measured Gas6 expression in the lungs of WT and IKKβ^ΔMye^ mice, which have myeloid cell-specific inhibition of NF-κB signaling resulting from targeted deletion of IKKβ [27]. At 1 week after urethane injection, we detected increased expression of Gas6 in whole lungs and in alveolar macrophages from IKKβ^ΔMye^ mice compared to WT mice (Figure 1C-1D). In addition, we assessed Gas6 expression by bone marrow-derived macrophages from WT and IKKβ^ΔMye^ mice after incubation with culture media from lung cancer (Lewis Lung Carcinoma, LLC) cells. Under these conditions, macrophages from WT mice expressed almost undetectable levels of Gas6 while Gas6 expression was induced in IKKβ^ΔMye^ macrophages (Figure 1E). Together, these data indicate that Gas6 expression is induced in tumor models and negatively regulated by NF-κB activation in macrophages.

### Reduction of Gas6 by warfarin treatment reduces lung tumorigenesis

**Figure 2:**
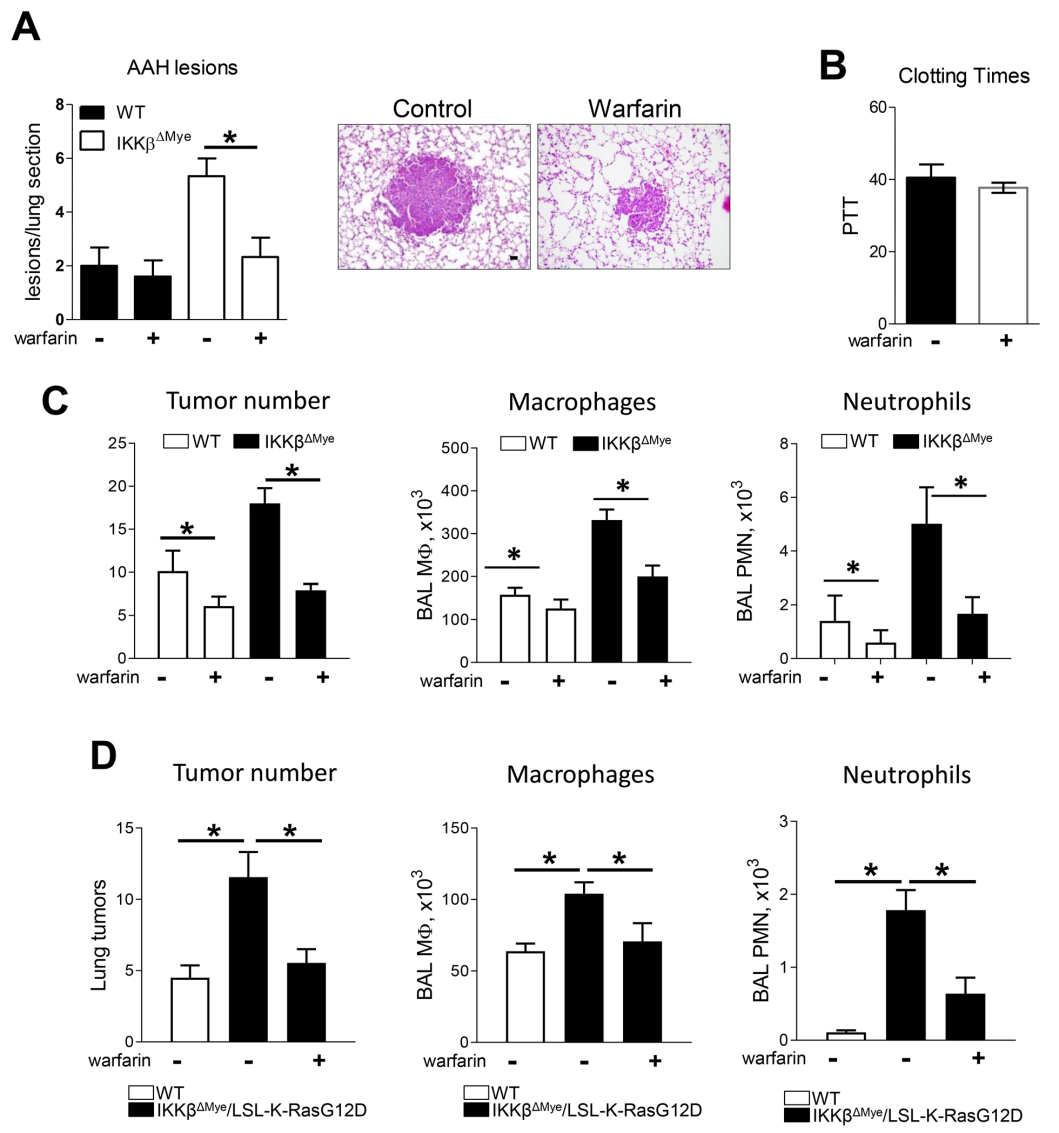
Warfarin treatment decrease lung tumor in urethane model and Kras spontaneous mouse tumor model of lung tumorigenesis **(A)** Pre-malignant atypical adenomatous hyperplasia (AAH) lesions number in lung sections from WT and IKKβ^ΔMye^ mice treated with or without warfarin in drinking water (250 mg/ml) for 6 weeks after urethane injection. Representative H&E staining of lungs isolated from WT and IKKβ^ΔMye^ mice after warfarin treatment showing lung tumors. **(B)** Blood clotting time in mice treated with warfarin. **(C)** Surface tumor number and number of cells in BAL at 4 months after urethane injection with or without warfarin treatment. **(D)** Lung tumors and number of cells in BAL from LSL-K-Ras^G12D^ mice that received bone marrow transplantation from WT or IKKβ^ΔMye^ mice. Lungs were harvested at 8 weeks after intratracheal instillation of Ad-Cre. Mean ± SEM, n=10 mice per group, ^*^p<0.05. Scale bars indicate 100 um.

To test whether Gas6 is involved in lung tumor promotion *in vivo*, we treated urethane-injected WT and IKKβ^ΔMye^ mice with the anticoagulant warfarin (0.25 mg/L) in drinking water as previously described [28–30]. Since Gas6 is a vitamin K dependent protein with a short half-life, low doses of warfarin that do not produce systemic anticoagulation can inhibit Gas6 production [31]. In this study, we detected increased numbers of atypical adenomatous hyperplasia (AAH) lesions, which are precursor lesions to lung adenomas, in the lungs of IKKβ^ΔMye^ mice at 6 weeks after urethane (Figure 2A). While warfarin treatment did not affect AAH lesions in WT mice, AAH lesions were reduced in warfarin-treated IKKβ^ΔMye^ mice to levels similar to those observed in WT mice (Figure 2A). This low dose of warfarin did not affect clotting time, indicating that this dose was insufficient to produce systemic anticoagulation (Figure 2B). At 4 months after urethane treatment, IKKβ^ΔMye^ mice had significantly increased tumor formation compared to WT mice, as we previously reported [27]; however, warfarin treatment substantially reduced lung tumorigenesis in both genotypes (WT and IKKβ^ΔMye^ mice) (Figure 2C). After warfarin treatment, tumor numbers were similar in lungs of WT and IKKβ^ΔMye^ mice. In addition to reduced tumor formation, warfarin treatment resulted in decreased macrophages and neutrophils in BAL at 4 months after urethane injection (Figure 2C).

In complimentary studies, we investigated a second lung cancer model driven by mutant K-Ras (LSL-K-Ras^G12D^ mice). For these studies, we performed bone marrow transplantation from WT and IKKβ^ΔMye^ mice to LSL-K-Ras^G12D^ mice, as previously reported [32]. After bone marrow reconstitution, we performed intratracheal instillation of Cre-expressing adenoviral vectors (Ad-Cre) to induce recombination, thereby allowing Ras^G12D^ expression in lungs. IKKβ^ΔMye^/LSL-K-Ras^G12D^ mice were then treated for 8 weeks with warfarin. Similar to the urethane model, tumor numbers were increased in IKKβ^ΔMye^/LSL-K-Ras^G12D^ mice compared to WT/LSL-K-Ras^G12D^ mice, and warfarin treatment blocked the increased tumorigenesis in IKKβ^ΔMye^/LSL-K-Ras^G12D^ mice (Figure 2D). Macrophages and neutrophils in BAL were decreased by warfarin treatment, similar to the urethane model (Figure 2D).

### MerTK signaling regulates tumorigenesis via increased proliferation

**Figure 3:**
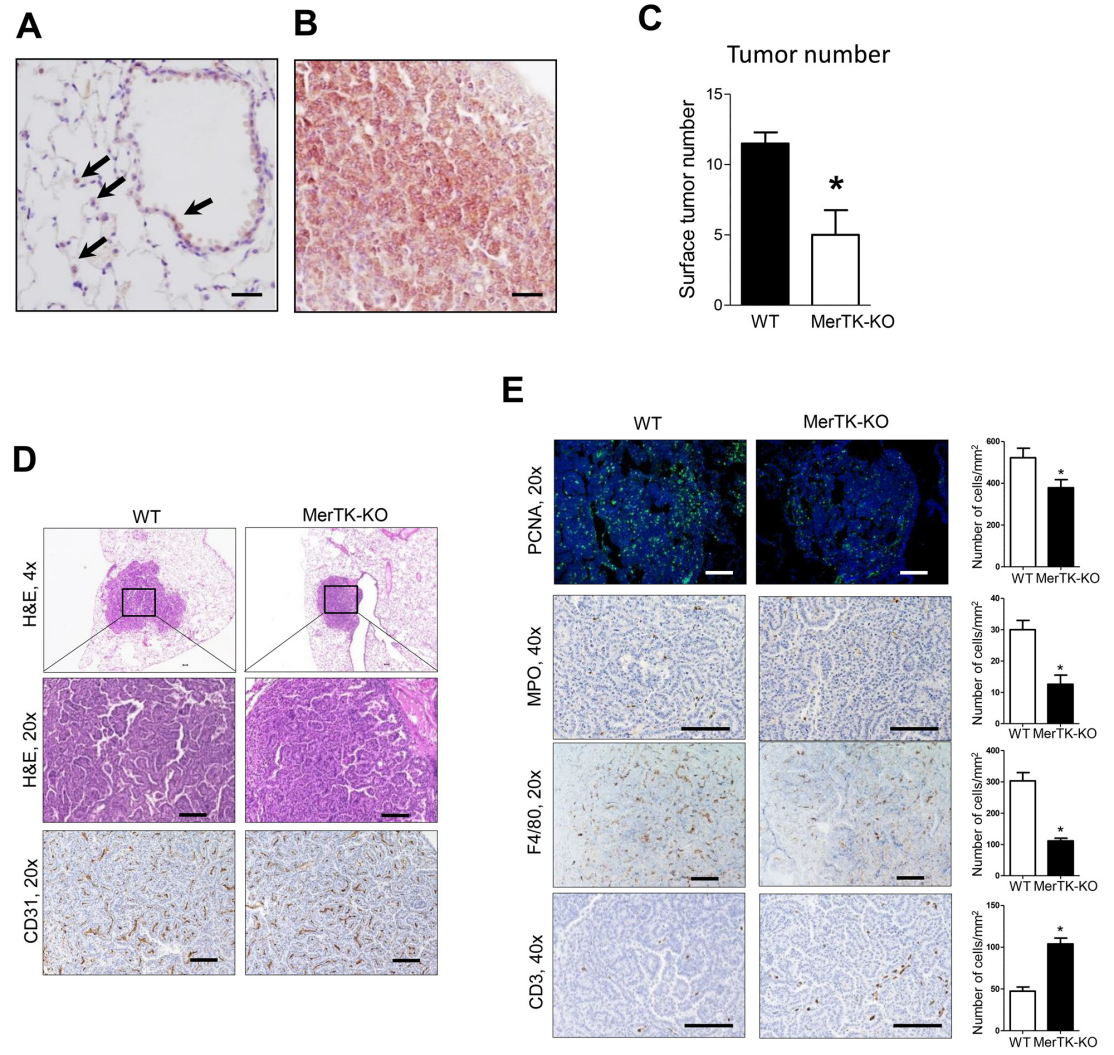
Reduced lung tumorigenesis in MerTK deficient mice **(A)** Expression of MerTK in normal airway epithelium and pulmonary macrophages (arrows) from WT mice. **(B)** Expression of MerTK in lung tumors from mice at 4 months after urethane injection. **(C)** Lung surface tumors at 4 months after urethane injection in WT and MerTK deficient mice. n=5 mice/group. **(D)** Representative H&E and CD31 immunostaining of lung tumors from WT and MerTK-KO mice **(E)** Identification and quantification of macrophages by F4/80 immunostaining, neutrophils by MPO immunostaining, T cells by CD3 immunostaining, and cell proliferation by PCNA immunofluorescence in lung tumors of WT and MerTK-KO mice. Cells were counted in tumors on 10 slides per lung from 5 mice/group. ^*^p<0.05. Scale bars indicate 100 um.

Of the TAMRs, MerTK has been shown to be expressed by a variety of tumor types, including non-small cell lung cancer (NSCLC) [16, 17, 24]. Therefore, we investigated whether this receptor could mediate the effects of Gas6 on lung tumorigenesis. By immunostaining, minimal MerTK staining was detected in alveolar epithelium and alveolar macrophages in normal lung (Figure 3A). However, as shown in Figure 3B, MerTK expression was markedly up-regulated in urethane-induced lung tumors. Lung tumorigenesis was significantly reduced in MerTK deficient mice at 4 months after urethane treatment (Figure 3C), suggesting that the Gas6-MerTK axis could be pro-tumorigenic in this model. Tumor histology and vascularity were similar between lung tumors from WT and MerTK deficient mice (Figure 3D); however, by PCNA immunofluorescence, tumor cell proliferation was significantly reduced in tumors from MerTK deficient mice (Figure 3E). In addition, we observed decreased macrophages and neutrophils in tumors from MerTK deficient mice, along with increased numbers of CD3+ T cells infiltrating the tumors (Figure 3E-3F).

**Figure 4:**
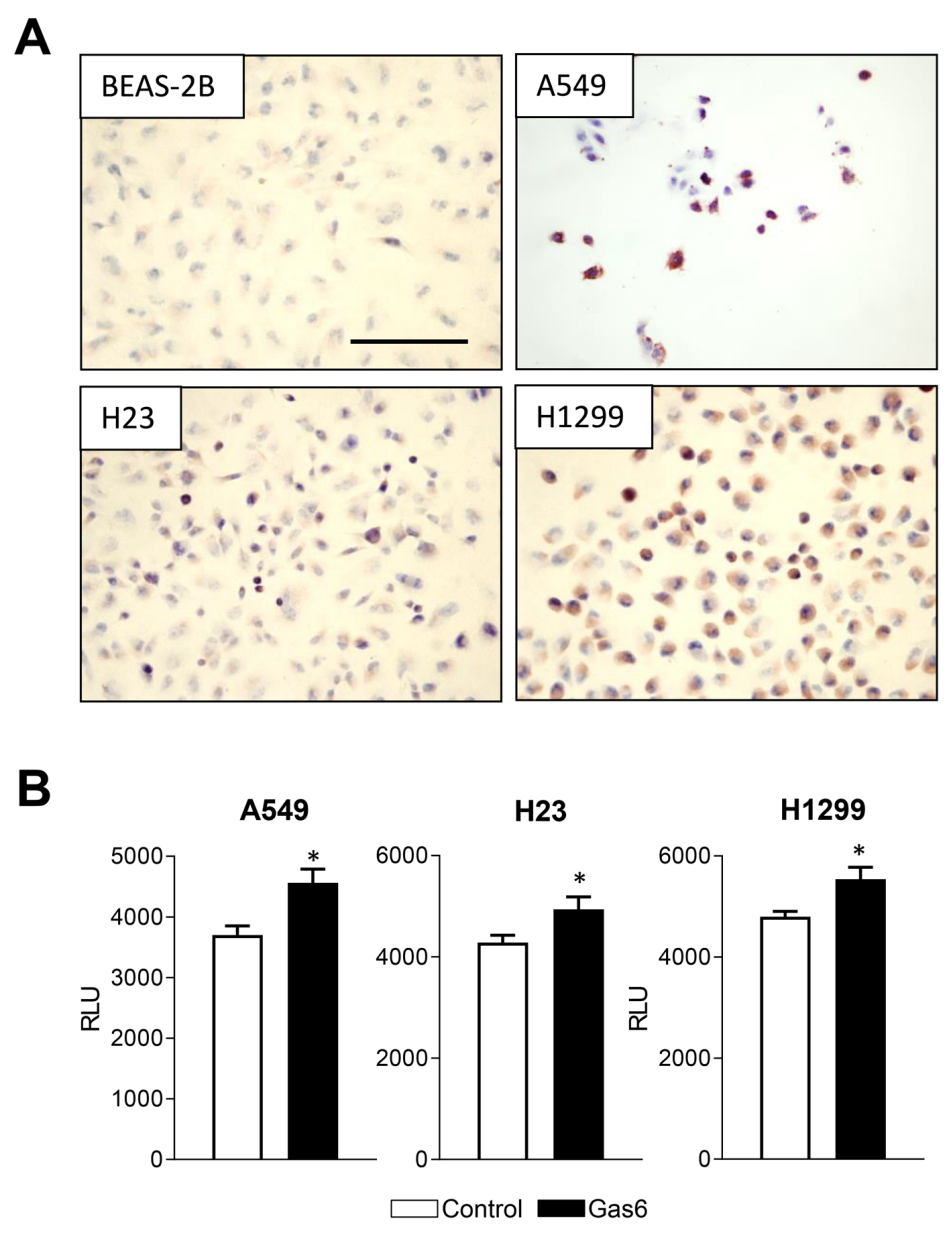
Gas6 promotes proliferation of human lung cancer cells **(A)** Immunocytochemistry for phospho-MerTK in normal human bronchial epithelial cells (BEAS-2B) and human lung tumor cell lines (H1299, H23, A549). **(B)** Proliferation of human lung tumor cell lines at 24 hours after addition of Gas6 (200 ng/ml) or vehicle. ^*^p<0.05. Scale bars indicate 100 um.

Next, we tested whether human NSCLC lines, including H23 cells (Kras mutation in codon 246), H1299 cells, (mutant Kras in codon 61), and A549 cells (Kras mutation in codon 12), along with non-malignant human bronchial epithelial cells (BEAS-2B), express functional MerTK. By immunostaining with phospho-MerTK antibodies, we found that H23, H1299, and A549 cells, but not BEAS-2B cells, showed highly active MerTK (Figure 4A). We then measured proliferation of these cell lines after treatment with Gas6 and found that Gas6 increased proliferation of all 3 cancer cell lines (Figure 4B) suggesting that the pro-tumor effects of Gas6-MerTK signaling are mediated via increased proliferation.

### The combination of MerTK inhibition and NF-κB inhibition effectively reduces tumor growth *in vivo*

**Figure 5:**
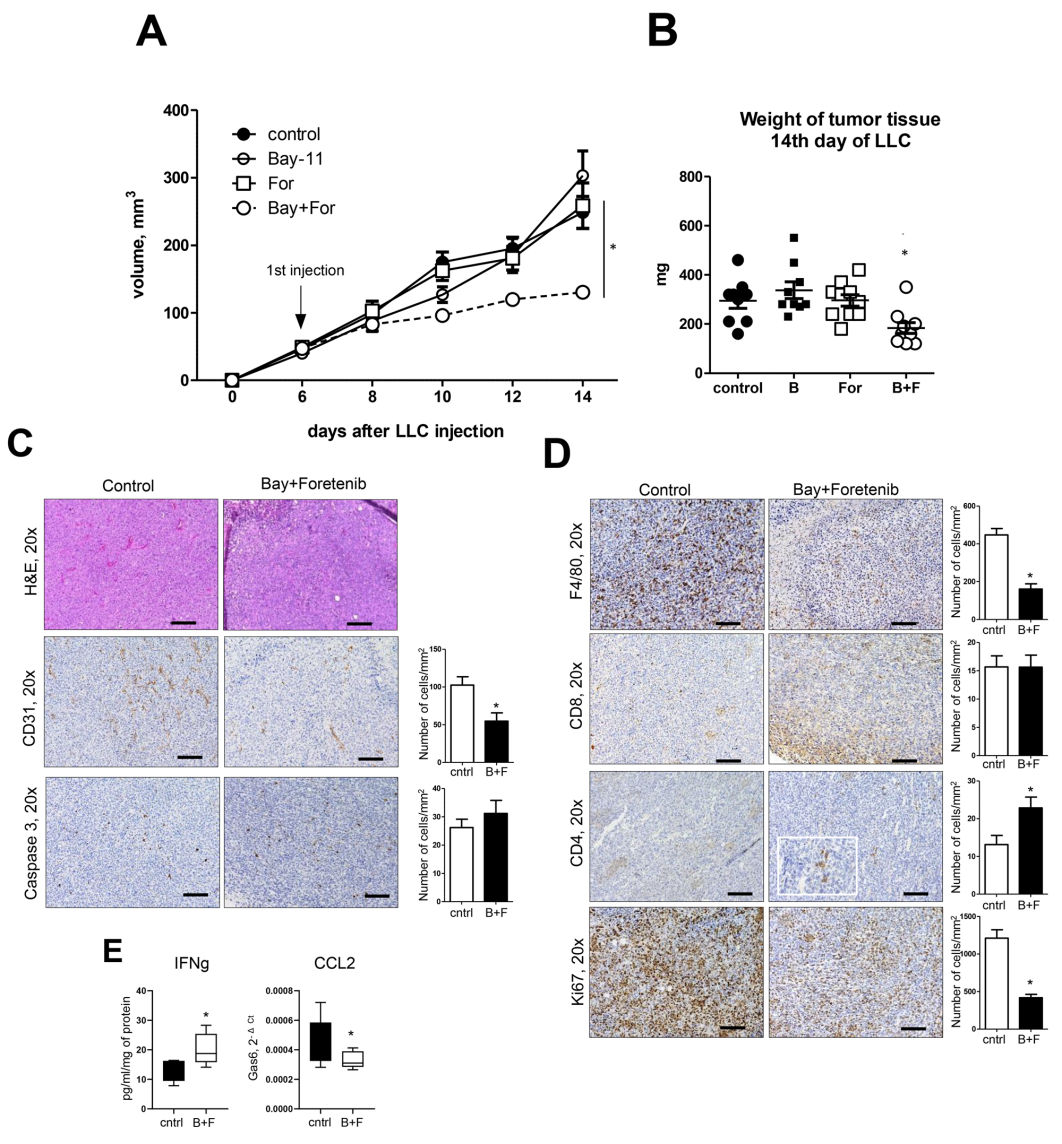
Treatment of mice with combination of NF-κB inhibitor and MerTK antagonist blocks lung cancer growth **(A)** Tumor size and **(B)** weight after subcutaneous injection of Lewis lung carcinoma (LLC) cells (5×10^5^/PBS). Mice were treated with Foretinib by gavage and/or Bay-117082 by intraperitoneal injection from day 6 until the day of harvest (day 14). (i.p). **(C, D)** H&E staining, and immunostaining for CD31, cleaved caspase 3, F4/80, CD4, CD8, and Ki-67 in representative tumors from control (vehicle treated) mice and mice treated with Foretinib + Bay-117082, Cells were counted in tumors on 10 slides per tissue from 10 mice/group. Scale bars indicate 100 um. **(E)** IFNγ levels in tissue homogenates from control mice and mice treated with Foretinib + Bay-117082 (B+F) and quantitative RT-PCR for CCL2 on RNA isolated from whole tumor tissue. n=10 mice per group. ^*^p<0.05.

To investigate the potential clinical utility of targeting MerTK in lung tumors with or without NF-κB inhibition, we utilized a heterotopic model in which tumors are induced by subcutaneous injection of LLC cells. After LLC cell injection, we treated mice with a MerTK inhibitor (Foretinib), an NF-κB inhbitior (Bay 11-70852), or both, beginning at day 6 after LLC injection (when the tumor size reached 50-100 mm^3^). Mice were euthanized at 14 days after LLC injection and tumors were examined. In comparison to vehicle-treated controls, no differences in tumor size or appearance were identified following treatment with Foretinib or Bay 11-70852 alone. In contrast, combination treatment resulted in a marked inhibition of tumor growth (Figure 5A). Tumor weights at Day 14 confirmed differences in tumor growth following combination treatment with Foretinib and Bay-117082 (Figure 5B). Immunohistochemistry studies showed that treatment with Foretinib and Bay-117082 resulted in fewer blood vessels and reduced macrophage infiltration of LLC tumors compared to other treatment groups (Figure 5C-5D). Tumor cell proliferation (by Ki-67 staining) was also reduced. In contrast, CD4^+^ T cells were increased in LLC tumors after treatment with Foretinib and Bay-117082, in parallel with an increase in interferon-γ (IFNγ) protein (Figure 5E). CD8+ T cells were not altered by combined treatment (Figure 5D). CCL2 expression was reduced by treatment with Foretinib and Bay-117082, consistent with the observed reduction in tumor macrophages (Figure 5E). Collectively, these findings indicate a strategy of combined treatment with MerTK and NF-κB inhibitors could be effective for reducing tumor cell proliferation, potentially by altering the composition of immune/inflammatory cells in the tumor microenvironment.

## DISCUSSION

Our study identified Gas6 and its receptor MerTK as a targetable, pro-tumorigenic pathway that contributes to resistance to NF-κB inhibitors in lung tumors. We found that lung macrophages are the primary source of Gas6 in lung cancer models and its production is inversely regulated by the NF-κB pathway. Blocking Gas6 protein production via warfarin treatment reduced lung tumorigenesis in two different lung cancer models, urethane and LSL-K-Ras^G12D^. Reduced lung tumors following warfarin treatment in bone marrow chimeras with IKKβ deficient myeloid cells in the LSL-K-Ras^G12D^ model further support a causative role for macrophage-produced Gas6 in the paradoxical increase in lung tumors found in these mice. MerTK deficient mice showed reduced lung tumor formation after urethane treatment, suggesting that this TAMR could be an important receptor for Gas6 in this model. Active MerTK was found in human lung cancer cell lines in culture and addition of Gas6 induced proliferation of these cells. To test the translational importance of these findings, we utilize an orthotopic tumor growth model and demonstrated that combined treatment with a MerTK inhibitor (Foretinib) and an NF-κB inhibitor, Bay-117082, substantially reduced tumor cell proliferation, vascularity, and tumor growth, whereas neither of these treatments alone was effective. In these experiments, we observed a beneficial change in the immune component in the tumor microenvironment with reduced macrophages and increased T cells. Together, these studies indicate that the Gas6-MerTK pathway limits the effectiveness of NF-κB inhibition in lung cancer development and progression, thus supporting wider use of combined targeted therapies for lung cancer.

Activation of the NF-κB pathway is connected with carcinogenesis by regulation of the inflammatory tumor microenvironment and plays an important role in malignant cell survival and proliferation [8]. Selective inhibition of NF-κB in airway epithelial cells reduces lung tumorigenesis in mice [9–12] and constitutive activation of NF-κB by over-expression of activated IKKβ results in 3-4-fold more lung tumors after carcinogen treatment [13]. Although these data point to inhibition of lung NF-κB as a potentially effective strategy for prevention or treatment of lung cancer, a variety of preclinical and clinical studies with NF-κB inhibitors have failed to show benefit [14, 15]. Previously, we suggested that the explanation for the apparent paradox that epithelial NF-κB inhibition reduces lung tumorgenesis while global NF-κB inhibition is ineffective lies in the effects of blocking NF-κB activity in immune inflammatory cells. This idea is supported by our finding that IKKβ^ΔMye^ mice with targeted NF-κB inhibition in myeloid cells have increased lung tumor formation [27]. In this prior study, we showed that neutrophil-derived IL-1β production mediates a portion of the increased tumor formation identified in IKKβ^ΔMye^ mice. Our current study extends work into the mechanisms of NF-κB resistance by identifying a role for macrophages via Gas6-MerTK signaling and suggesting that multiple immune/inflammatory cell populations could be involved in resistance to NF-κB inhibitors.

MerTK is a receptor tyrosine kinase of the TAM (Tyro3, Axl, MERTK) family, which has been reported to facilitate a number of cellular processes, including survival, proliferation, and differentiation [16, 33], through activation of PLCγ, PI3K, ERK and STAT pathways [34–36]. MerTK is expressed by many tumors, including NSCLC [16, 17, 24], and receptor blockade has been shown to reduce tumor growth [37], consistent with our findings. In addition, MerTK is a strong inducer of chemokines such as IL-8 and can activate FAK phosphorylation and recruitment to αvβ5 integrin, which is important for tumor cell migration [38].

Gas6 has a number of known functions in different cell types, including regulation of efferocytosis and polarization of macrophages [39]. Additionally, Gas6/MerTK can inhibit inflammation together with the interferon/STAT1 cascade by induction of SOCS1/SOCS3 expression. In the tumor microenvironment, Gas6 signaling can regulate a variety of pro-tumorigenic cellular functions and increased expression of Gas6 and MerTK predicts poor prognosis in many types of cancer [21]. Consistent with prior reports in other cancer models [25], we found that macrophages represent the main source of Gas6 in lung tumor models. Also, we showed that inhibiting the canonical NF-κB pathway in macrophages up-regulates Gas6 expression, thereby unmasking an important, pro-tumorigenic role for this pathway in lung cancer that appears to be mediated through increased tumor cell proliferation. While it remains unclear whether this inverse relationship between NF-κB and Gas6 represents direct transcriptional repression by NF-κB, increased Gas6 production and signaling appears to be an important mechanism of resistance to NF-κB inhibitors. Despite the inherent resistance to global NF-κB inhibition, targeted combination therapies, like NF-κB and Gas6/TAMR inhibition, could be effective for treatment of lung cancer.

## MATERIALS AND METHODS

### Animals, tumor models, drug administration

All animal care and experimental procedures were approved and conducted according to guidelines issued by the Vanderbilt University Institutional Animal Care and Use Committee. MerTK-KO mice on FVB background were provided by Dr. R. Cook (Vanderbilt University, Nashville, TN) [40]. Lung tumors were induced in IKKβ^Δmye^ mice (IKKβ^fl/fl^; LysM-Cre) [41] and littermate WT controls by a single intraperitoneal (i.p.) injection of urethane (ethyl carbamate, 1 g/kg) (Sigma-Aldrich). BAY 11-7082 (10 mg/kg body weight; Cayman Chemical) was delivered by i.p. injection as described previously [27]. Warfarin (250mg/L, Sigma) was delivered with drinking water. Lung tumors were induced in LSL-Kras^G12D^ mice [42], using intratracheal (i.t.) instillation of adeno-Cre (1.5 × 10^7^ plaque-forming units). Subcutaneous tumors in C57BL/6 mice were established by an injection of 5 × 10^5^ syngeneic LLC cells in 100 ul of PBS in the right flank. When tumor size reached about 3 mm in diameter (day 6), mice were randomized and treated with vehicle control, BAY 11-7082 (10 mg/kg, i.p.,), Foretinib (30 mg/kg, gavage), or a combination of BAY 11-7082 and Foretinib every second day. Tumor sizes were measured using Traceable digital calipers (Fisher Scientific). Bronchoalveolar lavage (BAL) cells were collected and counted as previously described [12].

### Histology and immunohistochemistry

Lung tumors and atypical adenomatous hyperplasia (AAH) lesions were counted as previously described [6]. For MER-TK detection lung sections or cell slides were immunostained with antibodies against PCNA (abcam) with secondary goat anti-mouse Alexa Flour 647 (Thermo Fisher), cleaved caspase-3 (Cell Signaling), CD3 (abcam), F4/80 (Novus Biologicals), CD31 (Dianova), CD4 (abcam), CD8 (abcam), Ki-67 (abcam). Staining was performed in Pathology Core an VUMC. Slides were placed on the Leica Bond Max IHC stainer. All steps besides dehydration, clearing and coverslipping are performed on the Bond Max. Slides are deparaffinized. Enzymatic induced antigen retrieval was performed using Proteinase K (Dako, Agilent, Santa Clara, CA) for 5 minutes. Slides were incubated with primary and later with secondary Ab. The Bond Polymer Refine detection system was used for visualization. Slides were then dehydrated, cleared and coverslipped.

### Clodronate macrophage depletion

Clodronate (Sigma) or PBS-containing liposomes were prepared as previously described [43]. 100 μL of clodronate or control PBS lipsomes was delivered intratracheal 3 days prior to DOX initiation, on the day of dox initiation, and weekly thereafter until mice were harvested at day 35. For liposome delivery, mice were anesthetized and intubated with a 1-mL syringe with a 6-mm-long, 22-gauge over-the-needle catheter (Abbocath-T; Venisystems). Macrophage depletion was confirmed by CD68 staining of lung sections. To obtain alveolar macrophages, BAL was performed by instilling four aliquots (0.8 ml) of sterile normal saline into the lungs with following 2hr incubation cells for harvesting only adherent cells [44].

### ELISA

IFNg in tumor tissue lysates was measured using the Mouse IFNg ELISA kits (R&D Systems, Minneapolis, MN) following the manufacturer’s protocol.

### Clotting assay

Plasma (35 μL) was mixed with PTT-A reagent (35 μL) followed by incubation for 5 minutes at 37°C. 18.75 mM CaCl_2_ (35 μL) was added, and time to clot formation was measured on an ST-4 Analyzer (Diagnostica Stago).

### Differentiation MF from bone marrow cells

Purified mouse bone marrow cells were cultured in RPMI 1640 medium containing 10% FBS (Invitrogen, Carlsbad, CA) at concentration of 5 × 10^6^/well in the presence of 10 ng/mL M-CSF (R&D Systems, Minneapolis, MN) in 3 ml of 6-well plate. The medium was replaced on 3^rd^ day and cells were treated on day 6 after isolation.

### Western blot

Whole lung lysates were prepared using CelLyticTM MT Cell Lysis Reagent (C3228; Sigma-Aldrich), separated by SDS-PAGE gel, transferred to nitrocellulose membrane, and probed with the anti-Gas6 (10AG2; R&D Systems) and anti-β-actin (A5316; Sigma-Aldrich). Immunodetection was performed using the corresponding AlexaFluor-conjugated antibodies and the Odyssey Infrared Imaging System (LI-COR Biosciences).

### Bone marrow transplantation

Lethally irradiated (9.5 Gy) recipient mice were injected with bone marrow cells (2 × 10^6^ bone marrow cells/mouse in PBS) from sex-matched, syngeneic donor mice. Animals were then housed for eight weeks under specific pathogen free (SPF) conditions with access to acidified water (pH 2.0) containing neomycin (100 mg/L, Sigma Aldrich) and polymyxin B (10 mg/L, Sigma-Aldrich) from 3 days before transplantation until 14 days after transplantation [32]. Mice were used for studies 8 weeks following transplantation.

### Real-time PCR

RNA from lung tissue or sorted myeloid cells was isolated using the RNeasy Mini kit (Qiagen). cDNA was generated using SuperScript III Reverse Transcriptase (Life Technologies) and subjected to Real-Time PCR using SYBR Green PCR Master Mix (Life Technologies). PCR primers are available by request. Relative mRNA expression in each sample was normalized to GAPDH and presented using the comparative Ct method (-2ΔCt).

### Proliferation

Cell viabilities were evaluated using MTT assay under different time points after treatment with Gas6 (200 ng/ml), 1 d, 3 d, 5 d, 7 d, and 9 d. Briefly, cells were seeded at density of 5000 in 96-well plates. After 48 h of culturing, 10 μL MTT solution (5 mg/mL) was added. Cells were then cultured for 4 h followed by removal of MTT solution. The supernatant was subsequently replaced with 180 μL DMSO. The optical density (OD) value was evaluated under 490 nm.

### Statistical analysis

Mouse data were analyzed using the GraphPad Prism 5.0 software (GraphPad Software), and values are presented as mean ± SEM. Pairwise comparisons were made using Student’s *t* tests. For experiments conducted over several time points or with multiple comparisons, a two-way ANOVA with a Bonferroni post-test was used.
